# SYD-1C, UNC-40 (DCC) and SAX-3 (Robo) Function Interdependently to Promote Axon Guidance by Regulating the MIG-2 GTPase

**DOI:** 10.1371/journal.pgen.1005185

**Published:** 2015-04-15

**Authors:** Yan Xu, Hidenori Taru, Yishi Jin, Christopher C. Quinn

**Affiliations:** 1 Department of Biological Sciences, University of Wisconsin-Milwaukee, Milwaukee, Wisconsin, United States of America; 2 Laboratory of Neuroscience, Graduate School of Pharmaceutical Sciences, Hokkaido University, Sapporo, Japan; 3 Division of Biological Sciences, Section of Neurobiology, and Howard Hughes Medical Institute, University of California San Diego, La Jolla, California, United States of America; Lunenfeld-Tanenbaum Research Institute of Mount Sinai Hospital, CANADA

## Abstract

During development, axons must integrate directional information encoded by multiple guidance cues and their receptors. Axon guidance receptors, such as UNC-40 (DCC) and SAX-3 (Robo), can function individually or combinatorially with other guidance receptors to regulate downstream effectors. However, little is known about the molecular mechanisms that mediate combinatorial guidance receptor signaling. Here, we show that UNC-40, SAX-3 and the SYD-1 RhoGAP-like protein function interdependently to regulate the MIG-2 (Rac) GTPase in the HSN axon of *C*. *elegans*. We find that SYD-1 mediates an UNC-6 (netrin) independent UNC-40 activity to promote ventral axon guidance. Genetic analysis suggests that SYD-1 function in axon guidance requires both UNC-40 and SAX-3 activity. Moreover, the cytoplasmic domains of UNC-40 and SAX-3 bind to SYD-1 and SYD-1 binds to and negatively regulates the MIG-2 (Rac) GTPase. We also find that the function of SYD-1 in axon guidance is mediated by its phylogenetically conserved C isoform, indicating that the role of SYD-1 in guidance is distinct from its previously described roles in synaptogenesis and axonal specification. Our observations reveal a molecular mechanism that can allow two guidance receptors to function interdependently to regulate a common downstream effector, providing a potential means for the integration of guidance signals.

## Author Summary

The nervous system is comprised of a complex network of axonal connections. This network is formed during development, when axons navigate to their target regions. Axon navigation requires multiple signaling pathways to detect and respond to extracellular guidance cues. Many of the guidance cues, receptors and signaling pathways have been identified. However, little is known about how the information encoded by different guidance cues is combined to arrive at a directional response. A key part of how this occurs is likely to involve combinatorial receptor signaling, when different guidance receptors work in combination with each other. However, the mechanisms that underlie combinatorial receptor signaling remain mostly unknown. In *C*. *elegans*, ventral axon guidance is mediated by the UNC-40 (DCC) and SAX-3 (Robo) guidance receptors. Here, we use genetic and biochemical analysis to identify a molecular mechanism that can mediate combinatorial signaling involving UNC-40, SAX-3 and the RhoGAP-like protein SYD-1.

## Introduction

Axonal migrations are guided through the developing nervous system by multiple extracellular guidance cues that activate receptors on the surface of the axonal growth cone. The axonal growth cone must integrate these multiple signals to arrive at a single directional response [1–7]. Progress has been made in characterizing individual guidance cues, receptors and their signaling pathways. However, the signaling mechanisms that allow for integration between receptor signaling pathways are largely unknown [8].

Integration of multiple guidance signals could be mediated by combinatorial receptor signaling [8]. In fact, several examples of combinatorial axon guidance receptor signaling have been identified in various systems. For example, commissural axons in the mammalian spinal cord exhibit a hierarchical interaction between the DCC (UNC-40) and Robo (SAX-3) receptors [9]. Upon reaching the midline, spinal axons are exposed to Slit, which promotes formation of a DCC/Robo heterodimer that silences the attractive response to Netrin, thereby allowing the axons to exit the midline. Variations of hierarchical interactions between the DCC and Robo receptors have also been observed in several other systems [10–13]. Moreover, hierarchical interactions between semaphorin receptors have also been observed [14,15].

Guidance signaling pathways can also exhibit synergistic interactions. For example, in *C*. *elegans*, the AVM neuron is simultaneously exposed to both the UNC-6 (Netrin) and SLT-1 (Slit) guidance cues. Both of these cues guide the AVM axon towards the ventral nerve cord. UNC-40 (DCC) can function individually as a receptor for UNC-6, whereas SAX-3 (Robo) can function individually as a receptor for SLT-1. However, UNC-40 can also function independently of UNC-6, when it binds to the SAX-3 receptor to promote the response to SLT-1 [16]. Despite these observations, the signaling mechanisms that mediate combinatorial guidance signaling remain mostly unexplored.

SYD-1 is an intracellular protein that contains a RhoGAP-like domain that has been implicated in synaptogenesis and axonal specification [17–21]. Here, we present evidence that SYD-1 can also function in axon guidance to promote UNC-40 signaling independently of its canonical ligand UNC-6. Our data suggest that UNC-40, SAX-3 and SYD-1 can function interdependently to regulate the MIG-2 GTPase. Our observations provide an example of a signaling mechanism that can mediate combinatorial guidance signaling.

## Results

### Interactions between the UNC-6 and SLT-1 signaling pathways

**Fig 1:**
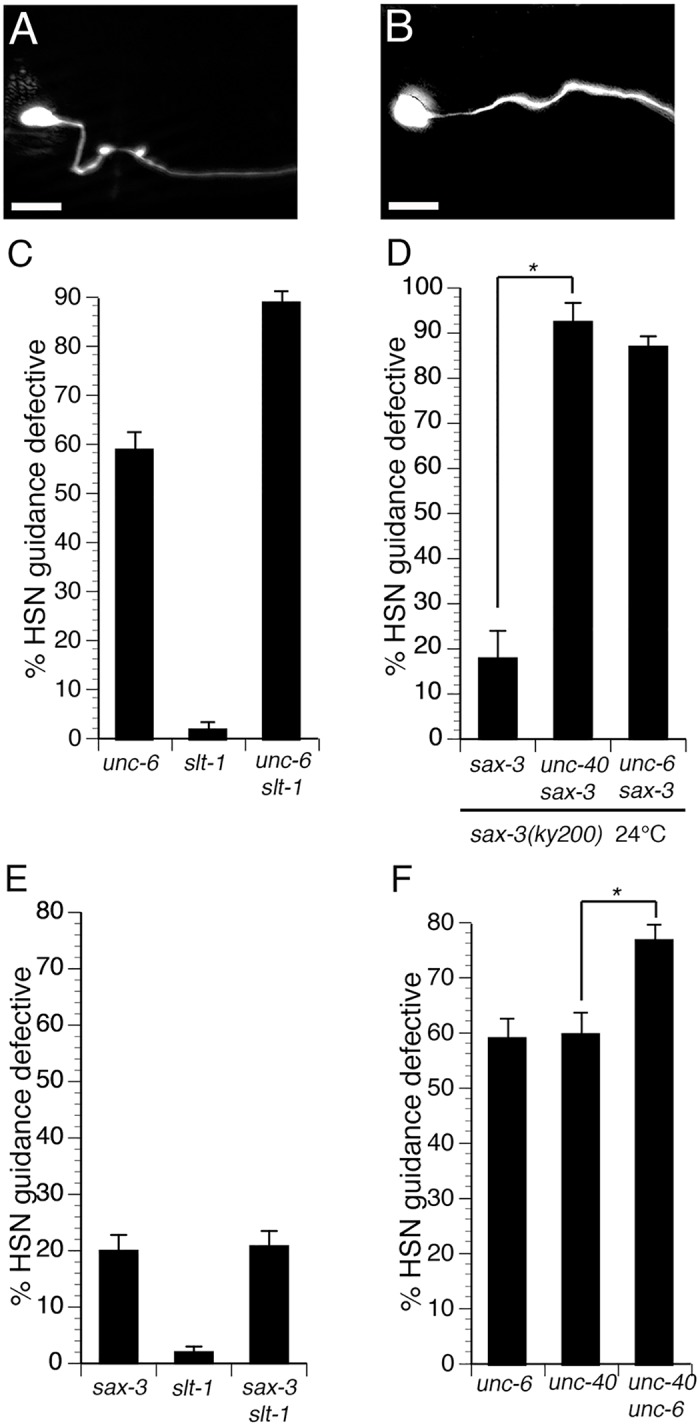
Interactions between the UNC-6 and SLT-1 signaling pathways in the HSN neuron. (A) Example of normal HSN axon guidance. (B) Example of defective HSN axon guidance. (C) Synergistic genetic interaction between null mutations in the genes that encode the UNC-6 and SLT-1 guidance cues. (D) The UNC-40 and SAX-3 pathways can function in parallel to each other. For the data shown in this panel, the *sax-3(ky200)* temperature sensitive allele was used to circumvent lethality. (E) The SAX-3 receptor can function independently of the SLT-1 ligand. A null mutation in the gene that encodes the SLT-1 guidance cue results in only rare guidance defects. However, a null mutation in the gene that encodes SAX-3, the receptor for SLT-1, results in 20% guidance defects. Guidance defects are not enhanced in *sax-3 slt-1* double null mutants. (F) The UNC-40 receptor can function independently of the UNC-6 guidance cue. A null mutation in the gene that encodes the UNC-40 receptor causes HSN guidance defects at a penetrance that is similar that caused by a null mutation in the gene that encodes the UNC-6 guidance cue. However, loss of UNC-40 function enhances guidance defects in the absence of UNC-6 function, indicating that UNC-40 can function independently of UNC-6. For the data shown in panel D, we analyzed progeny of worms maintained at 20°C and shifted to 24° just prior to egg laying. For all other panels, strains were grown at 20°C. Scale bars are 10 μm. Anterior is to the right. Ventral is down. Alleles shown in all panels, except where noted in panel D, are *unc-6(ev400)*, *slt-1(eh15)*, *sax-3(ky123)*, *unc-40(e1430)*. HSN axon guidance was scored as defective if the axon failed to reach the ventral nerve cord. For the data shown in panel D, n>39. For all other data, n≥200. Brackets indicate statistically significant difference, Z test for proportions (***p<0.0001, **p<0.001).

The cell body of the HSN neuron is located on the lateral body wall of *C*. *elegans* and extends an axon ventrally to the ventral nerve cord. To determine the relative contributions of guidance cues in HSN ventral axon guidance, we examined HSN axon guidance in *slt-1(eh15)* and *unc-6(ev400)* null mutants (Fig 1A–1C). We found that the *unc-6* null mutants exhibited 59% guidance defects, whereas the *slt-1* null mutants had 2% guidance defects. However, the *unc-6 slt-1* double null mutants had 89% guidance defects. These observations suggest that the HSN axon is guided primarily by the UNC-6 and SLT-1 guidance cues. Moreover, the synergistic nature of these interactions suggest interactions between the UNC-6 and SLT-1 signaling pathways in the HSN neuron, providing a system for studying the factors that mediate interactions between these pathways.

To investigate interactions between the signaling pathways that transduce the UNC-6 and SLT-1 cues, we examined guidance defects in mutants that disrupt their receptors, UNC-40 and SAX-3, respectively. We attempted to construct an *unc-40; sax-3* double null mutant. However, this double null mutant was completely embryonic lethal, precluding analysis of axon guidance. As an alternative we used the *sax-3(ky200)* temperature sensitive mutation. The single *sax-3(ky200)* mutant at 24°C has guidance defects with a penetrance of 19%, suggesting that this allele behaves as a strong loss of function at 24°C (Fig 1D). We constructed an *unc-40(e1430); sax-3(ky200)* double mutant. Although these animals were mostly embryonic lethal at 24°C, we were able to analyze axon guidance in the survivors and found that guidance defects in *unc-40(e1430); sax-3(ky200)* double mutants were significantly enhanced relative to either single mutant (Fig 1D). Likewise, we also found that guidance defects in *unc-6(ev400) sax-3(ky200)* double mutants were significantly enhanced relative to either single mutant. These results suggest that UNC-40 and SAX-3 can function in parallel to each other and that these two receptors mediate most, if not all, of the ventral guidance signals in the HSN axon.

SAX-3 can function independently of its canonical ligand SLT-1. To investigate the relationship between SLT-1 and SAX-3, we analyzed mutants that disrupt these proteins. Although SAX-3 can function as a receptor for the SLT-1 guidance cue [22–24], we found that in the HSN, *sax-3(ky123)* null mutants had 20% defects, whereas *slt-1(eh15)* null mutants had only 2% defects (Fig 1E). The *sax-3 slt-1* double mutants showed no enhancement relative to the *sax-3* single mutants. These observations suggest that in addition to being a receptor for SLT-1, SAX-3 can also function independently of SLT-1. These results are consistent with several previous findings of SLT-1 independent SAX-3 function [12,23,25].

UNC-40 can function independently of its canonical ligand UNC-6. UNC-40 primarily functions as a receptor for the UNC-6 guidance cue [26–27]. However, the penetrance of HSN axon guidance defects was enhanced in *unc-40(e1430); unc-6(ev400)* double null mutants relative to *unc-40(e1430)* and *unc-6(ev400)* single null mutants (Fig 1F). Similar results were obtained using the *unc-40(n324)* null allele (S1 Fig). These observations suggest that UNC-40 can function independently of UNC-6, in the HSN neuron, consistent with previous reports of an UNC-6 independent function for UNC-40 in several different processes [16,26,28–30]. Moreover, the identification of an UNC-6 independent UNC-40 signaling activity in the HSN neuron provides a means to identify the signaling mechanisms that mediate interactions between the UNC-40 and the SAX-3 signaling pathways.

### SYD-1 mediates UNC-6 independent UNC-40 signaling

**Fig 2:**
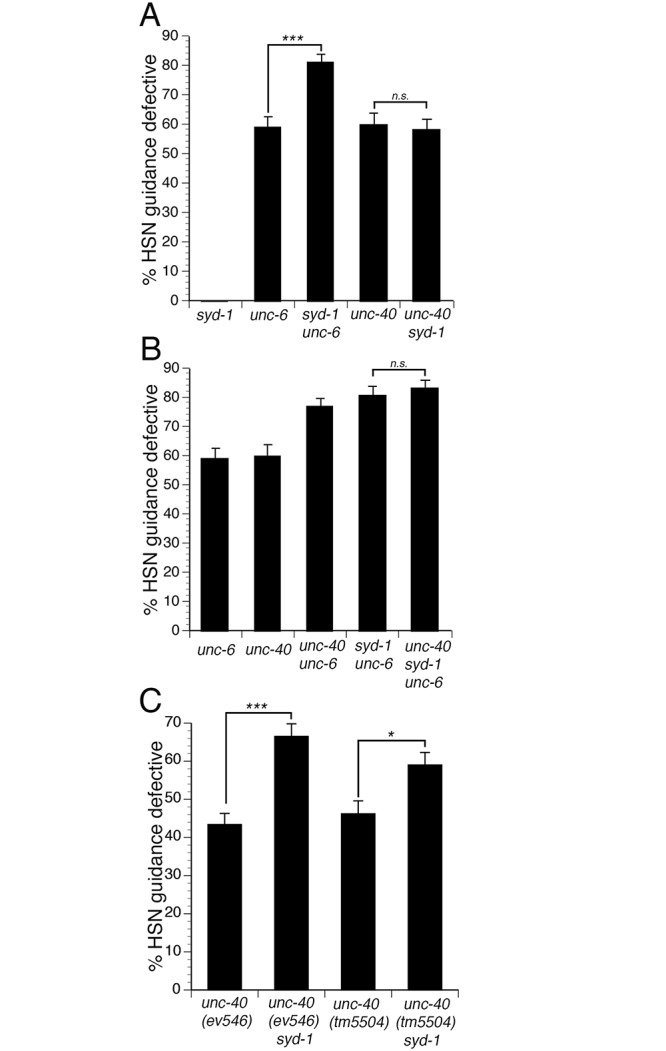
SYD-1 mediates UNC-6 independent UNC-40 signaling. (A) SYD-1 functions in parallel to UNC-6, but not UNC-40. The *syd-1(ju2)* null mutation enhances HSN guidance defects in *unc-6* null mutants. However, the *syd-1* null mutation does not enhance guidance defects in an *unc-40* null mutant. (B) SYD-1 functions with UNC-40 independently of UNC-6. Guidance defects in an *unc-40*; *syd-1; unc-6* triple null mutant are not enhanced relative to *unc-40*; *unc-6* or *syd-1*; *unc-6* double null mutants. (C) The *syd-1* null mutation enhances HSN guidance defects in *ev546* and *tm5504* hypomorphic *unc-40* mutants. Alleles for panels A and B are *unc-6(ev400)*, *syd-1(ju2)*, *unc-40(e1430)*. Alleles for panel C are *unc-40(ev546)*, *unc-40(tm5504)* and *syd-1(ju2)*. HSN axon guidance was scored as defective if the axon failed to reach the ventral nerve cord. For all experiments, n≥200. Brackets indicate statistically significant difference, Z test for proportions (***p<0.0001, *p<0.01).

**Fig 3:**
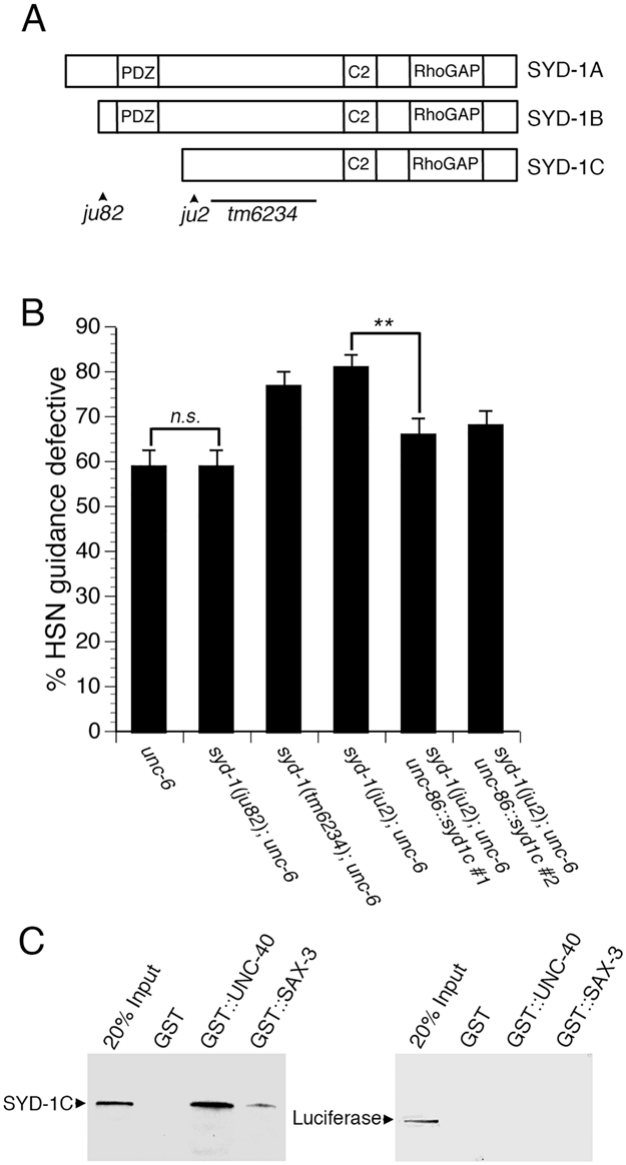
The function of the *syd-1* gene in axon guidance is mediated by the SYD-1C isoform. (A) Schematic of the three isoforms of SYD-1 and location of three mutations. The *ju82* mutation is a nonsense mutation the affects the SYD-1A and SYD-1B isoforms. The *ju2* mutation is a nonsense mutation that affects all three isoforms. The *tm6234* mutation is a deletion that affects all three isoforms. (B) The SYD-1C isoform is necessary and sufficient to mediate the role of SYD-1 in axon guidance. Guidance defects in *unc-6* null mutants are enhanced by the *ju2* and *tm6234* mutations, but not by the *ju82* mutation. Guidance defects in a *syd-1*; *unc-6* double null mutant are rescued by expressing SYD-1C in the HSN neuron with an *unc-86*::*syd-1c* transgene. (C) SYD-1C binds to the cytoplasmic domain of UNC-40 fused to GST (GST::UNC-40) and also to the cytoplasmic domain of SAX-3 fused to GST (GST::SAX-3), but not to GST alone. An unrelated protein, luciferase, does not bind to GST, GST::UNC-40, or GST::SAX-3. For reference, an amount of protein (SYD-1C or luciferase) equal to 20% of the amount of protein in the binding assay was run on the gel (20% Input). HSN axon guidance was scored as defective if the axon failed to reach the ventral nerve cord. For all experiments, n≥200. Bracket indicates statistically significant difference, Z test for proportions (**p<0.001).

To identify a signaling mechanism that can mediate UNC-6 independent UNC-40 function, we searched for candidate mutations that can enhance guidance phenotypes in *unc-6* null mutants, but not in *unc-40* null mutants. We found that the *syd-1(ju2)* null mutation could enhance guidance defects in *unc-6(ev400)* null mutants, but not in *unc-40(e1430)* null mutants (Fig 2A). We also obtained similar results using different null alleles for *syd-1* and *unc-40* (Figs 3B and S2). These observations suggest that SYD-1 might mediate UNC-6 independent UNC-40 signaling.

To determine if SYD-1 and UNC-40 function together to mediate UNC-6 independent UNC-40 function, we analyzed guidance defects in *unc-40*; *syd-1; unc-6* triple null mutants and compared these to defects in the *syd-1*; *unc-6* and *unc-40*; *unc-6* double null mutants (Fig 2B). If *unc-40* and *syd-1* function in a genetic pathway, the triple mutant should have a penetrance of guidance defects similar to the double mutants. Indeed, we found that the penetrance of guidance defects in the *unc-40*; *syd-1*; *unc-6* triple mutant was similar to the *syd-1*; *unc-6* or the *unc-40*; *unc-6* double null mutants. These observations are consistent with a model where UNC-40 and SYD-1 function together to mediate UNC-6 independent UNC-40 function.

To further test the idea that UNC-40 and SYD-1 function together, we analyzed double mutants between the *syd-1(ju2)* null allele and two different hypomorphic *unc-40* alleles (Fig 2C). When two genes function in a pathway, a loss of function mutation in one gene can enhance defects associated with a hypomorphic mutation in the second gene. Indeed, we found that guidance defects associated with either of two *unc-40* hypomorphic alleles could be enhanced by the *syd-1(ju2)* null mutation, consistent with the idea that *unc-40* and *syd-1* function in a genetic pathway.

### Guidance function of SYD-1 is mediated by isoform C

SYD-1 is expressed as three different isoforms, known as SYD-1A, SYD-1B, and SYD-1C. SYD-1A and SYD-1B contain a PDZ domain, whereas SYD-1C does not contain the PDZ domain (Fig 3A). Loss of SYD-1A and SYD-1B disrupts axonal identity in DD and VD motor neurons [17] and also disrupts presynaptic development in the HSN neuron [20,31]. For these phenotypes, the *syd-1(ju82)* mutation that disrupts SYD-1A and SYD-1B, but spares SYD-1C (see Fig 3A), behaves as a null allele. These observations indicate that SYD-1C is not sufficient for axonal specification or presynaptic development.

To examine the role of each isoform of SYD-1 in axon guidance, we analyzed guidance defects in different mutant alleles of *syd-1* (Fig 3B). The *syd-1(ju82)* allele disrupts the SYD-1A and SYD-1B isoforms, but not the SYD-1C isoform. The *syd-1(ju2)* and *syd-1(tm6234)* alleles disrupt all three isoforms. We found that the *syd-1(ju82)* allele, did not enhance guidance defects in *unc-6* null mutants. However, both *syd-1(ju2)* and *syd-1(tm6234)* did enhance guidance defects in *unc-6* null mutants. These genetic interactions suggest that the role of the *syd-1* gene in guidance may be mediated by the SYD-1C isoform. To further test the role of the SYD-1C isoform, we used the *unc-86* promoter to drive expression of SYD-1C in the HSN neuron. We found that expression of SYD-1C in the HSN neuron can rescue guidance defects in *syd-1(ju2)*; *unc-6(ev400)* double null mutants (Fig 3B), indicating that the SYD-1C isoform is sufficient to mediate the function of the *syd-1* gene in guidance. Moreover, these results also indicate that SYD-1C functions cell-autonomously in the HSN neuron. Together, these results suggest that the protein domains contained within SYD-1C are sufficient to mediate axon guidance and that the PDZ domain contained within SYD-1A and SYD-1B is not required for axon guidance. By contrast, loss of SYD-1A and SYD-1B disrupts synaptogenesis and axon specification, suggesting that the PDZ domains are required for these functions and implying that the function of SYD-1 in guidance is distinct from its role in synaptogenesis and axon specification. Although our data suggest that the SYD-1C isoform is necessary and sufficient to mediate the role of *syd-1* in axon guidance, other interpretations are possible and we cannot rule out potential roles for the other isoforms of SYD-1.

### The cytoplasmic domains of UNC-40 and SAX-3 bind to SYD-1C

Since genetic data suggest that UNC-40 and SYD-1 function together, we tested for a physical interaction between SYD-1C and the cytoplasmic domain of UNC-40 (Fig 3C). We found that SYD-1C binds to the cytoplasmic domain of UNC-40 fused to GST (GST::UNC-40), but not to GST alone. In addition, we found that SYD-1C can also bind to the cytoplasmic domain of SAX-3 (GST::SAX-3), but not to GST alone. By contrast, we observed no binding between an unrelated protein, luciferase, and either GST::UNC-40 or GST::SAX-3. These observations suggest that SYD-1C can bind to the cytoplasmic domains of both UNC-40 and SAX-3.

### SYD-1 binds to and negatively regulates the MIG-2 GTPase

**Fig 4:**
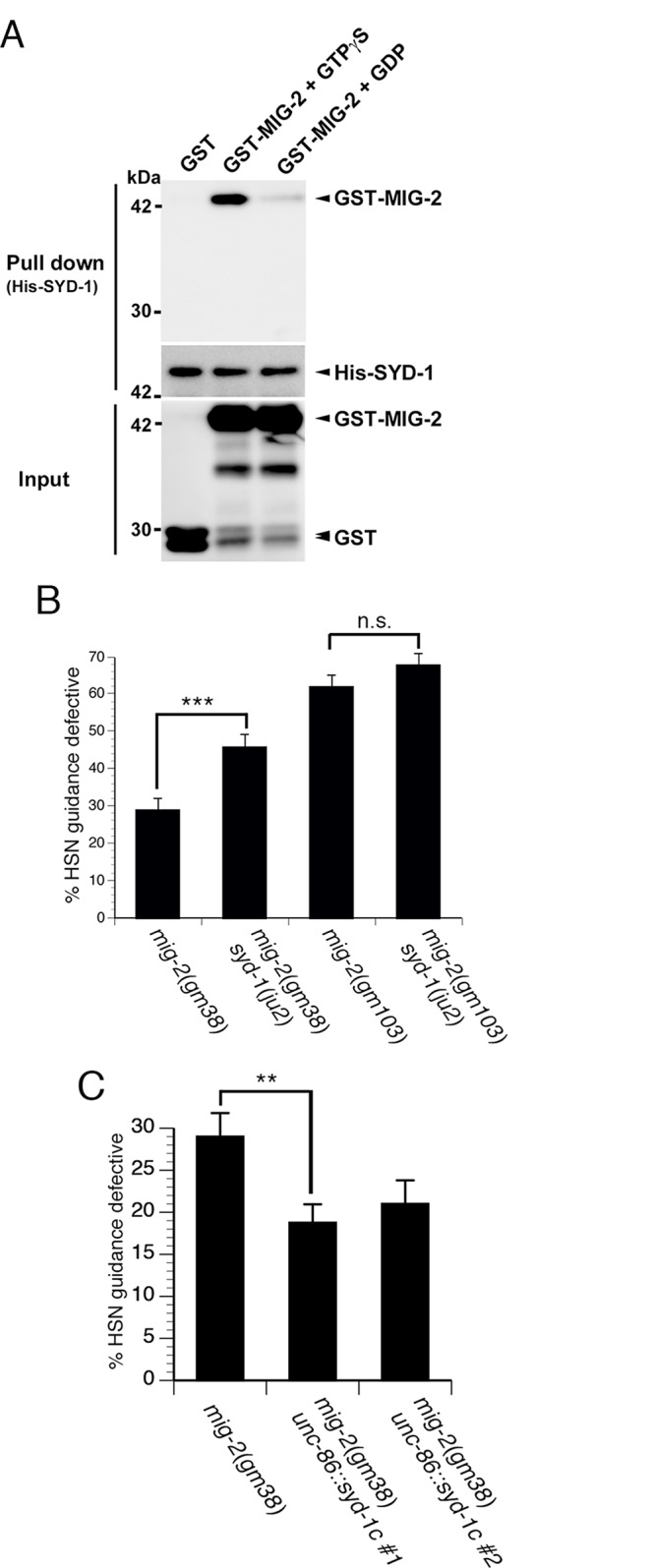
SYD-1 promotes axon guidance by negatively regulating the MIG-2 GTPase. (A) The RhoGAP-like domain of SYD-1 can bind to the active form of MIG-2. Binding between the C-terminal RhoGAP-like domain of SYD-1 (His-SYD-1) and GST-MIG-2 was substantially enhanced after incubation with GTPγS (GST-MIG-2 + GTPγS) relative to incubation with GDP (GST-MIG-2 + GDP). No binding was observed between His-SYD-1 and GST. (B-C) SYD-1 negatively regulates MIG-2 activity. (B) The *syd-1(ju2)* null mutation enhances HSN guidance defects in partially activated *mig-2(gm38)* mutants. However, the null mutation in *syd-1* does not enhance HSN guidance defects in the fully activated *mig-2(gm103)* mutants. (C) Transgenic expression of SYD-1C suppresses axon guidance defects in the partially activated *mig-2(gm38)* mutants. HSN axon guidance was scored as defective if the axon failed to reach the ventral nerve cord. For all experiments, n≥200. Brackets indicate statistically significant difference, Z test for proportions (***p<0.0005, **p<0.005).

SYD-1 contains a RhoGAP-like domain, but has not been associated with any Rho GTPases. By testing candidate GTPases, we found that the GAP-like domain of SYD-1 binds to MIG-2. To confirm this interaction we performed binding assays with recombinant MIG-2 and the C-terminus of SYD-1, which contains the RhoGAP-like domain (Fig 4A). We found that MIG-2 fused to GST (GST-MIG-2) was pulled-down with the His6-tagged C-terminus of SYD-1 (His-SYD-1), while GST alone was not. Moreover, binding between MIG-2 and SYD-1 was markedly enhanced when MIG-2 was bound to GTPγS (a non-hydrolyzable analog of GTP) relative to when MIG-2 was bound to GDP. These observations indicate that the RhoGAP-like domain of SYD-1 preferentially binds to the GTP-bound active form of MIG-2.

**Fig 5:**
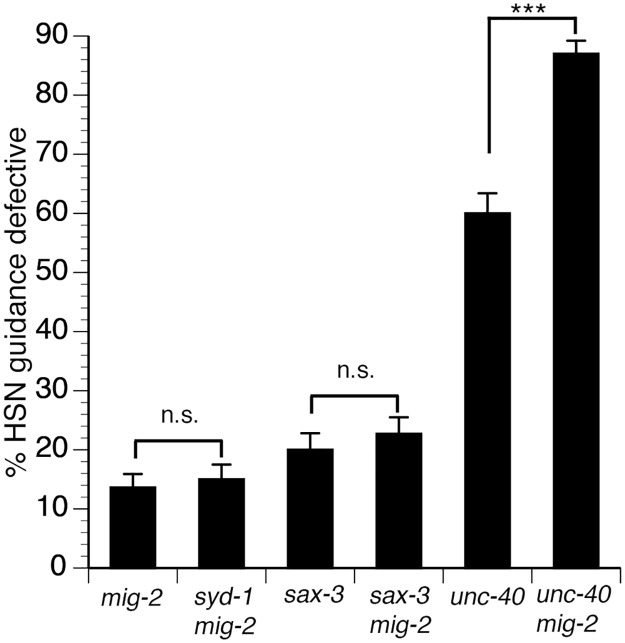
MIG-2 functions with SAX-3 to regulate axon guidance. HSN guidance defects were observed in *mig-2* null mutants and in *sax-3* null mutants. HSN guidance defects were not enhanced in *sax-3 mig-2* double null mutants, indicating that SAX-3 and MIG-2 function together to regulate HSN axon guidance. The *mig-2* null mutant enhances HSN guidance defects in *unc-40* null mutants. Alleles are *syd-1 (ju2)*, *mig-2(ok2273)*, *sax-3(ky123)*, *unc-40(e1430)*. HSN axon guidance was scored as defective if the axon failed to reach the ventral nerve cord. For all experiments, n≥200. Brackets indicate statistically significant difference, Z test for proportions (***p<0.0001).

To determine if SYD-1 can regulate MIG-2 activity, we tested for genetic interactions between the *syd-1(ju2)* null allele and *mig-2* gain of function mutations. The *mig-2(gm103)* mutation encodes a fully activated MIG-2, whereas *mig-2(gm38)* encodes a partially activated MIG-2 [32–33]. We found that loss of *syd-1* can enhance the defects associated with the partially active *mig-2(gm38)* allele (Fig 4B), suggesting that SYD-1 can negatively regulate MIG-2. By contrast, we observed no enhancement of the fully active *mig-2(gm103)* allele, consistent with the expectation that fully activated MIG-2 cannot be further activated. To further test the functional relationship between SYD-1 and MIG-2, we transgenically expressed SYD-1C in the *mig-2(gm38)* partially activated gain of function mutants (Fig 4C). Consistent with the idea that SYD-1 can negatively regulate MIG-2, we found that transgenic expression of SYD-1C suppresses guidance defects caused by the *mig-2(gm38)* gain of function mutation. To further test the interaction between SYD-1 and MIG-2, we constructed a *syd-1(ju2)*; *mig-2(ok2273)* double null mutant. We found that penetrance of HSN guidance defects in these double null mutants was not enhanced relative to *mig-2(ok2273)* single mutants (Fig 5), further supporting the idea that SYD-1 functions with MIG-2. Together, these data are consistent with a model where SYD-1 can negatively regulate the activation state of MIG-2.

### MIG-2 functions in the SAX-3 signaling pathway

To consider the role of *mig-2* in HSN axon guidance, we examined the HSN axon in *mig-2(ok2273)* null mutants (Fig 5). We found that 14% of HSN axon migrations were defective in the *mig-2* null mutants, indicating that MIG-2 is required for HSN axon guidance. Likewise, 20% of HSN axon migrations were defective in *sax-3(ky123)* null mutants. To determine if *mig-2* functions in a genetic pathway with *sax-3*, we examined *sax-3 mig-2* double null mutants. If two genes function in a genetic pathway, a double null mutant should have defects similar to the greatest of the single mutants. We found that the *sax-3 mig-2* double mutants had a penetrance of guidance defects similar to that of *sax-3* single mutants, indicating that *mig-2* and *sax-3* do function in a genetic pathway. Consistent with a function for *mig-2* in the *sax-3* pathway, we also found that the *mig-2* null mutation can enhance defects in the *unc-40* null mutants. Together, these genetic interactions support the conclusion that MIG-2 functions in the SAX-3 pathway.

### SYD-1 function requires SAX-3

**Fig 6:**
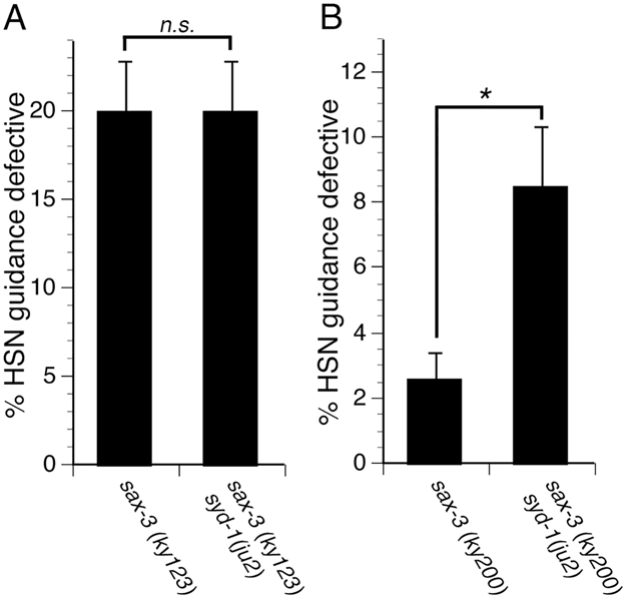
SYD-1 function requires SAX-3. (A) The *syd-1(ju2)* null mutation does not enhance guidance defects in *sax-3(ky123)* null mutants. (B) The *syd-1(ju2)* null mutation enhances guidance defects in *sax-3(ky200)* hypomorphic mutants. For experiments with the *sax-3(ky123)* null mutants, we scored only severe HSN defects (the axon never reaches the ventral nerve cord). For the *sax-3(ky200)* hypomorphic mutants, we did not observe any severe HSN axon guidance defects, but only mild defects (the axon trajectory is defective but eventually reaches the ventral nerve cord). Therefore, for experiments with the *sax-3(ky200)* hypomorphic mutants, we scored mild axon guidance defects. For all experiments, n≥200. Bracket indicates statistically significant difference, Z test for proportions (*p<0.01).

Since SYD-1 regulates MIG-2, which functions with SAX-3, we asked if SYD-1 function requires SAX-3. To address this question, we examined genetic interactions between mutations in *syd-1* and *sax-3*. We found that the *syd-1(ju2*) null mutation fails to enhance axon guidance defects in *sax-3(ky123)* null mutants, suggesting that SYD-1 might function with SAX-3 (Fig 6A). To further determine if SYD-1 can function with SAX-3, we used the *sax-3(ky200)* hypomorphic allele and found that the *syd-1(ju2)* null mutation enhances guidance defects in *sax-3(ky200)* hypomorphic mutants (Fig 6B). Since, the *syd-1* null mutant can enhance guidance defects in the *sax-3* hypomorphic mutants, but not in *sax-3* null mutants, we conclude that SYD-1 functions with SAX-3, implying that SYD-1 function requires SAX-3.

## Discussion

### The UNC-40 and SAX-3 pathways can function separately or in combination with each other

We have found that single null mutants of *unc-40* and *sax-3* exhibit partially penetrant guidance defects. We have also found that *unc-40*; *sax-3* double strong loss of function mutants exhibit guidance defects that are nearly fully penetrant. Together, these results suggest that UNC-40 and SAX-3 can function in parallel to each other.

Although UNC-40 and SAX-3 can function in parallel, our genetic analysis suggests that UNC-40 and SAX-3 can also function together. This idea is consistent with previous work in the AVM neuron showing that UNC-40 and SAX-3 can function in combination with each other, whereby UNC-40 functions independently of UNC-6 to potentiate SAX-3 signaling [16]. Consistent with this genetic analysis, biochemical data indicate that both UNC-40 and SAX-3 can bind to each other [16]. Likewise, DCC and Robo, vertebrate homologs of UNC-40 and SAX-3, respectively, also exhibit functional and biochemical interactions [9–11]. Despite these findings, little is known about the signaling events that mediate combinatorial guidance receptor function.

### UNC-40, SAX-3 and SYD-1 function interdependently

Our results suggest that SYD-1 function requires both UNC-40 and SAX-3, implying that UNC-40, SAX-3 and SYD-1 function interdependently. Loss of *syd-1* function fails to enhance guidance defects in null mutants of either *unc-40* or *sax-3*. However, loss of sy*d-1* function does enhance guidance defects in hypomorphic mutants of either *unc-40* or *sax-3*. These observations suggest that SYD-1 function does not occur in the complete absence of UNC-40 or SAX-3, but becomes apparent when UNC-40 or SAX-3 function is reduced. Moreover, we also find that SYD-1 binds to the cytoplasmic domains of both UNC-40 and SAX-3. Together, these observations are consistent with a model whereby SYD-1 does not function in the UNC-40 individual pathway or the SAX-3 individual pathway, but rather functions in a common pathway with both UNC-40 and SAX-3. However, we note that other interpretations for these results are possible and that future investigations will be required to further define the relationships between UNC-40, SAX-3 and SYD-1.

### Models for UNC-40 and SAX-3 combinatorial signaling

Our results suggest that UNC-40, SAX-3 and SYD-1 function interdependently, implying that SYD-1 can mediate combinatorial receptor signaling. We propose two models to explain how UNC-40, SAX-3 and SYD-1 could mediate combinatorial signaling (Figs S3 and S4). In the heterodimer model, UNC-40 and SAX-3 would bind to each other and function as an UNC-6 independent guidance receptor. This heterodimer receptor would bind to SYD-1 and cause negative regulation of MIG-2, which functions in the SAX-3 pathway. Alternatively, in the crosstalk model, UNC-40 and SAX-3 would not be physically associated with each other. In this crosstalk model, SYD-1 would bind to the cytoplasmic tail of the UNC-40 receptor and would negatively regulate MIG-2, which functions in the SAX-3 pathway. We favor the heterodimer model because previous results have indicated that UNC-40 and SAX-3 can bind to each other [16], our genetic analysis suggest that SYD-1 function requires both UNC-40 and SAX-3, and our biochemical analysis show that SYD-1 can bind to both UNC-40 and SAX-3. Future investigations may help differentiate between these two models, or potentially suggest other models.

### SYD-1 can negatively regulate the MIG-2 GTPase

Our finding that a *syd-1* null allele can enhance guidance defects in partially activated *mig-2* mutants, but not in fully activated *mig-2* mutants, implies that SYD-1 is involved in regulating the activation state of MIG-2. However, SYD-1 lacks two critical residues that are required for GAP activity [34–35]. Thus far, attempts to detect SYD-1 GAP activity have been unsuccessful. Since we have found that the GAP domain of SYD-1 can bind to activated MIG-2, we propose that SYD-1 functions as a scaffold that can promote negative regulation of MIG-2 activity. One possibility is that SYD-1 and MIG-2 may be part of a complex that also includes a GAP protein. Alternatively, it remains possible that SYD-1 could possess GAP activity on its own.

### Axon guidance signaling requires both positive and negative regulation of Rac GTPases

We report that UNC-6 independent UNC-40 signaling is mediated by SYD-1 and that SYD-1 negatively regulates MIG-2. Thus, UNC-6 independent UNC-40 signaling can promote axon guidance by negatively regulating MIG-2. This idea is consistent with previous results showing that negative regulation of Rac promotes signaling downstream of Robo (SAX-3). In *Drosophila*, CrossGAP (Vilse) has been identified as a GAP that negatively regulates Rac activation to promote Robo signaling [36,37]. In mammalian neurons, srGAP1 has been identified as a GAP for Rac and Cdc42 that is required for Robo signaling [38–39]. Positive regulation of Rac has also been reported, whereby Son of Sevenless (Sos), a Guanine Nucleotide Exchange Factor (GEF), can positively regulate Rac to promote Robo signaling [40]. Thus, Robo signaling involves both positive and negative regulation of Rac. Consistent with this idea, guidance defects result from either Rac gain of function or Rac loss of function mutations [32,41]. Likewise, guidance defects are caused by loss of function or gain of function in CrossGAP, a GAP for Rac in the *Drosophila* [36–37]. Together, these observations indicate that axon guidance requires precise control of Rac activation state, which is achieved through both positive and negative regulation of Rac.

### SYD-1C and its mammalian homolog, mSYD1A, contain predicted N-terminal Intrinsically Disordered Domains

SYD-1C resembles its mammalian homolog mSYD1A, in that both are predicted to contain an Intrinsically Disordered Domain (ID domain) at their N-terminus, rather than a PDZ domain [42]. ID domains can shift between disordered and ordered confirmations and can also engage in intramolecular interactions that affect the function of other domains [42,43]. Although SYD-1C and mSYD1A both contain predicted ID domains, the amino acid sequence of the ID domain is not conserved between SYD-1C and mSYD1A. Consistent with this different amino acid sequence, the function of SYD-1C and mSYD1A appears to be different, in that the former regulates axon guidance and the later regulates synaptic vesicle docking [42]. These different functions are likely to be mediated by different binding partners for the ID domains of SYD-1C and mSYD1A.

### Evidence that the SAX-3 receptor can function independently of its canonical ligand SLT-1

Although our study has focused on UNC-6 independent UNC-40 function, we have also observed evidence for SLT-1 independent SAX-3 function. This idea is supported by the higher penetrance of HSN guidance defects in *sax-3* null mutants relative to *slt-1* null mutants. In fact, SLT-1 independent SAX-3 signaling has been observed in several different processes, including AVM and PVM axon guidance [12,23,25,44]. Much like in the HSN, the PVM neuron shows a higher penetrance of HSN guidance defects in *sax-3* null mutants relative to *slt-1* null mutants [12]. Genetic analysis has suggested that in the PVM neuron SAX-3 can inhibit UNC-40 signaling in the absence of SLT-1 [12]. This inhibitory interaction between SAX-3 and UNC-40 also occurs in the AVM neuron [12,44]. However, the AVM neuron shows a higher penetrance of guidance defects in *slt-1* null mutants relative to *sax-3* null mutants. The reason for the differences in *slt-1* and *sax-3* mutant defects in the AVM relative to the HSN and PVM are not known. However, these differences could be explained by a greater degree of redundant signaling in the HSN and PVM neurons relative to the AVM neuron.

### Evidence that the UNC-6 guidance cue can function independently of its canonical receptor UNC-40

Since we have found that UNC-40 can function independently of UNC-6, one might expect *unc-40* null mutants to have a greater penetrance of guidance defects relative to *unc-6* null mutants. However, we have found that *unc-6* and *unc-40* null mutants have a similar penetrance of HSN guidance defects. One possible explanation could be that UNC-6 can also function independently of UNC-40 in the HSN. Indeed, the existence of UNC-40 independent UNC-6 activity is apparent in AVM and PVM axon guidance, where *unc-6* mutants have a higher penetrance of guidance defects relative to *unc-40* mutants [12]. Moreover, ENU-3 is thought to form part of an UNC-40 independent UNC-6 signaling pathway in the AVM and PVM neurons [45].

## Materials and Methods

### *C*. *elegans* genetics

Genetic manipulations were carried out using standard procedures. The N2 Bristol genetic background was used in all experiments and all animals were maintained at 20°C on NGM plates seeded with OP50 bacteria. The following alleles were used and are considered to be null: *unc-6(ev400)* [46], *slt-1(eh15)* [26], *sax-3(ky123)* [47], *unc-40(e1430)* [26], *syd-1(ju2)* [17], *syd-1(tm6234)*, *syd-1(ju82)* [17], *mig-2(ok2273)* [48]. The following alleles are considered to be hypomorphic: *sax-3(ky200)* [47], *unc-40(ev546)* [49], *unc-40(tm5504)* [49]. The following alleles are considered to be gain of function: *mig-2(gm38)* [32], *mig-2(gm103)* [32]. The *unc-40(ev546)* allele was obtained from Joseph Culotti. The *syd-1(tm6234)* allele was obtained from Shohei Mitani. All other alleles were obtained from the *Caenorhabditis* Genetics Center (CGC).

### DNA constructs

The pAGC5 plasmid includes Punc-86::syd1c::sl2::tagrfp and was created using Gibson Assembly and cloned into pCFJ910, provided by Erik Jorgensen via Addgene (Addgene Plasmid #44481). The DNA sequence encoding TAGRFP was obtained from Julie Plastino [50]. pRSET-SYD-1C (pCZGY658), which encodes His6-tagged C-terminus of SYD-1C protein (AA320-721), was generated using the Gateway cloning system (Invitrogen).

### Transgenic rescue

Transgenes expressing SYD-1C in the HSN neuron were created by injecting the pAGC5 plasmid at 4 ng/μl with 50 ng/μl of odr-1::RFP and 50 ng/μl Bluescript into wildtype worms. Two independent transgenic lines were established and crossed into the syd-1(ju2); unc-6(ev400) genetic background. HSN axon guidance was analyzed as described below.

### Analysis of phenotypes

Axon guidance was analyzed as described previously [51,52]. The zdIs13 transgene [53], which expresses Ptph-1::gfp, was used to visualize the HSN axon in young adult animals. Except where noted, HSN guidance defects were scored as defective if the axon failed to reach the ventral nerve cord. For experiments involving the sax-3(ky200) hypomorphic allele, only weak defects were observed, where the axon trajectory is abnormal, but the axon eventually reaches the ventral nerve cord. Therefore, in experiments involving the sax-3(ky200) hypomorphic allele, HSN axons were scored as defective if their trajectory was abnormal, but they eventually reached the ventral nerve cord.

### GST binding assays with GST::MIG-2

Recombinant GST and GST-MIG-2 proteins were produced in *Escherichia coli* DH5α with plasmid pGEX6P and pGEX4T-MIG-2 (obtained from Hiroshi Qadota [54], respectively, by incubating for 5 h at 37°C in the presence of 0.3 mM IPTG. His6-tagged C-terminus of SYD-1C containing RhoGAP-like domain (His-SYD-1) was produced in *E*. *coli* BL21(DE3) with pRSET-SYD-1C (pCZGY658) by incubating for 16 h at 22°C in the presence of 0.3 mM IPTG. GST- and His6- tagged proteins were extracted by sonication in PBS pH 7.4 with 0.5% Triton X-100 and protease inhibitors (Roche) and centrifuging. GST-MIG-2 was incubated with 0.1 mM of GTPγS or 1 mM of GDP (Sigma-Aldrich) in PBS pH 7.4 with 0.5% Triton X-100, 10 mM MgCl_2_, 10 mM EDTA, 2% glycerol and protease inhibitors for 30 min at 30°C, and then supplied additional MgCl_2_ to 75 mM. For pull-down analysis, His-SYD-1 was immobilized to HisPur Cobalt Resin (Thermo), incubated with GST or GST-MIG-2 proteins for 1 h at 4°C, washed with PBS pH 7.4 with 0.1% Triton X-100 and 20 mM imidazole for 4 times, eluted with 300 mM imidazole in PBS pH 7.4, 0.1% Triton X-100. The eluate and input were subjected to SDS-PAGE, and GST- and His- tagged proteins were detected by western blotting with mouse monoclonal Anti-GST antibody (Upstate Biotechnology) and Anti-His-tag mAb (Medical and biological laboratories), respectively, using ECL Advance Western Blotting Detection Kit (GE Healthcare).

### Binding assays with GST::UNC-40 and GST::SAX-3

SYD-1C protein was produced with the TNT SP6 quick coupled *in vitro* transcription and translation system (Promega) and labeled with biotin. GST::UNC-40 was produced in bacteria and coupled to Glutathione-Sepharose. Binding assays were conducted for 16 hours at 4°C in PBS with 0.1% Triton X-100, 1% BSA and protease inhibitors. After binding, samples were washed 3 times with wash buffer (PBS and 0.1% Triton X-100). Bound material was detected by SDS-PAGE electrophoresis and western blotting with Strepavidin-Alkaline Phosphatase.

## Supporting Information

S1 Fig The *unc-40(n324)* mutation enhances axon guidance defects associated with the *unc-6(ev400)* mutation. HSN axon guidance was scored as defective if the axon failed to reach the ventral nerve cord. For all experiments, n≥200. Brackets indicate statistically significant difference, Z test for proportions (***p<0.0001).
(TIF)

S2 Fig Guidance defects associated with *unc-40* null mutants are not enhanced by mutations in *syd-1*. The *syd-1(tm6234)* mutation is predicted to affect all three isoforms of SYD-1 and does not enhance guidance defects associated with the *unc-40(e1430)* null mutation. The *syd-1(ju2)* null mutation is expected to affect all three isoforms of SYD-1 and does not enhance guidance defects associated with the *unc-40(n324)* null mutation. HSN axon guidance was scored as defective if the axon failed to reach the ventral nerve cord. For all experiments, n≥200.
(TIF)

S3 Fig Two models for how UNC-40, SAX-3 and SYD-1 can function interdependently to regulate MIG-2. (A) In the heterodimer model, UNC-40 and SAX-3 form a heterodimer that interacts with SYD-1. In this model UNC-40, SAX-3 and SYD-1 function together to negatively regulate MIG-2. MIG-2 can also be activated by SAX-3. (B) In the cross talk model, UNC-40 and SAX-3 are not physically associated. UNC-40 associates with SYD-1 and negatively regulates MIG-2, which functions in the SAX-3 pathway.
(TIF)

S4 Fig Model describing how guidance receptors can function in individual or combined pathways. UNC-40 can function individually by activating effectors such as CED-10, MIG-10, UNC-34 and UNC-115. SAX-3 can function individually by activating MIG-2. UNC-40 and SAX-3 can also function in a combined pathway, where they collaborate to regulate MIG-2. SYD-1 is specific to the combined pathway. This example is depicted with the heterodimer model (see S3 Fig), however the same idea could also apply to the cross talk model.
(TIF)
