# Supplementary figures and images for: SYD-1C, UNC-40 (DCC) and SAX-3 (Robo) Function Interdependently to Promote Axon Guidance by Regulating the MIG-2 GTPase
Source: PLoS Genet. 2015 Apr 15;11(4):e1005185. doi: 10.1371/journal.pgen.1005185 (PMC4398414; doi:10.1371/journal.pgen.1005185)

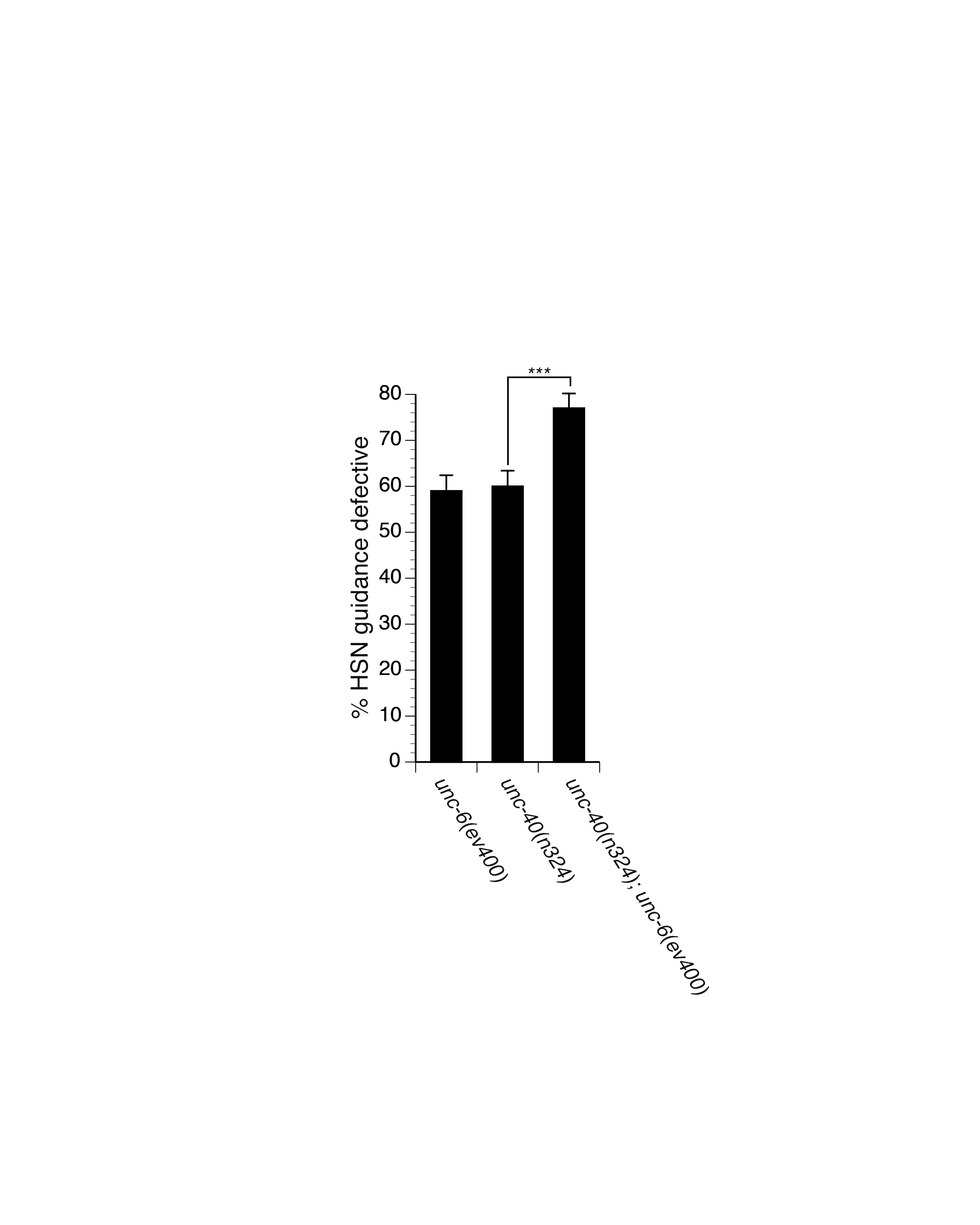

Supplement: S1 Fig — HSN axon guidance was scored as defective if the axon failed to reach the ventral nerve cord. For all experiments, n≥200. Brackets indicate statistically significant difference, Z test for proportions (***p<0.0001). (TIF) [file pgen.1005185.s001.tif]

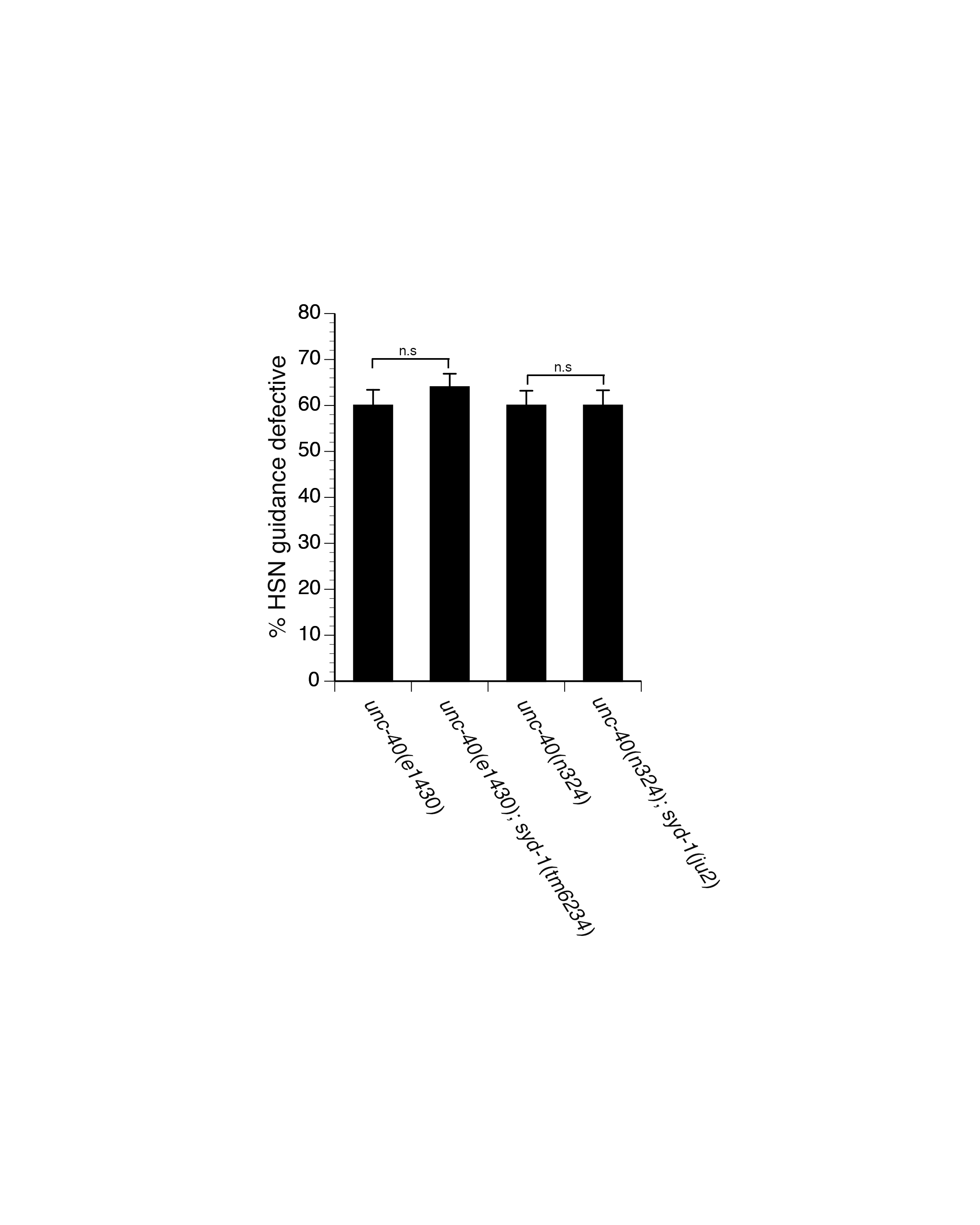

Supplement: S2 Fig — The syd-1(tm6234) mutation is predicted to affect all three isoforms of SYD-1 and does not enhance guidance defects associated with the unc-40(e1430) null mutation. The syd-1(ju2) null mutation is expected to affect all three isoforms of SYD-1 and does not enhance guidance defects associated with the unc-40(n324) null mutation. HSN axon guidance was scored as defective if the axon failed to reach the ventral nerve cord. For all experiments, n≥200. (TIF) [file pgen.1005185.s002.tif]

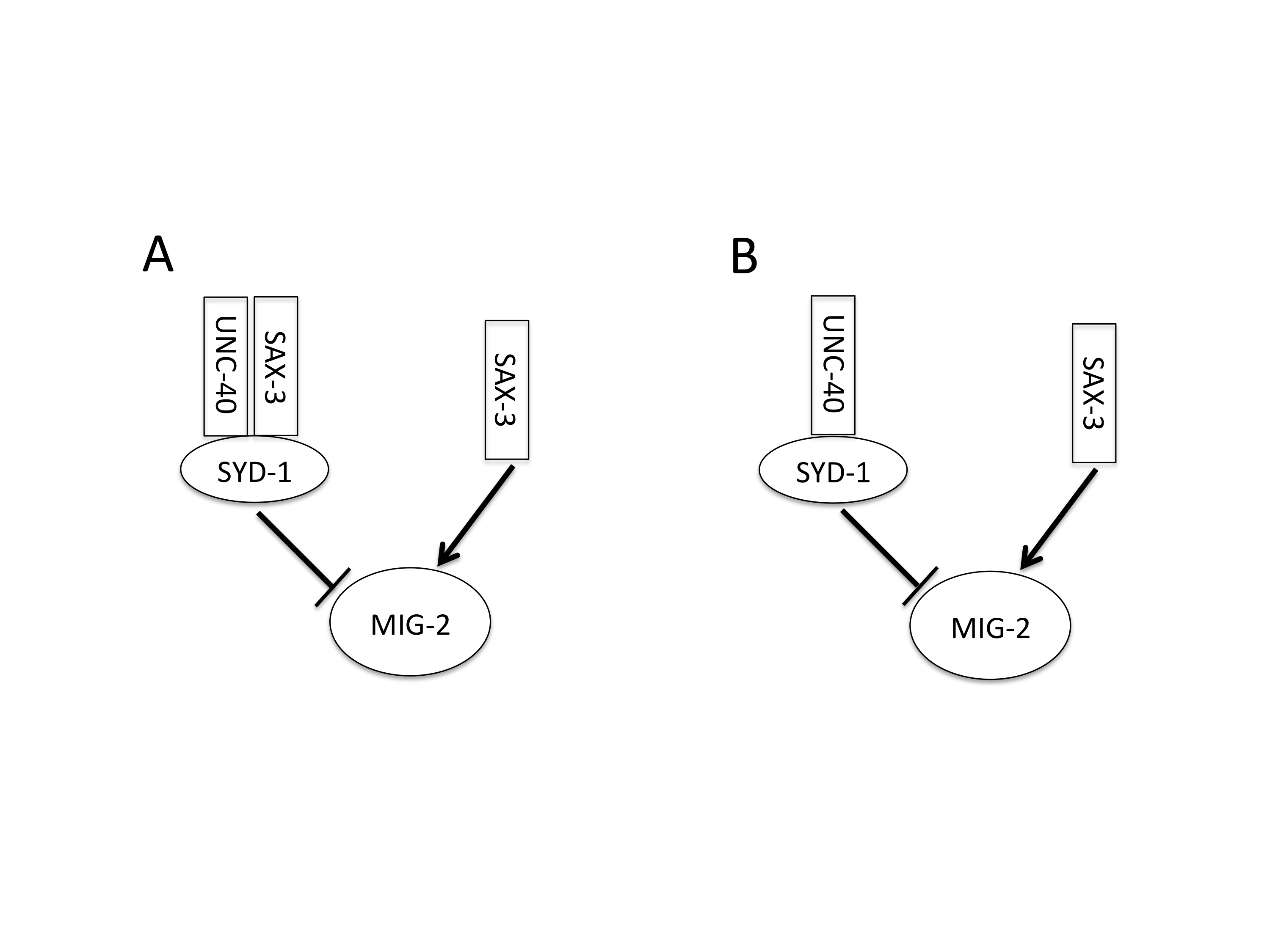

Supplement: S3 Fig — (A) In the heterodimer model, UNC-40 and SAX-3 form a heterodimer that interacts with SYD-1. In this model UNC-40, SAX-3 and SYD-1 function together to negatively regulate MIG-2. MIG-2 can also be activated by SAX-3. (B) In the cross talk model, UNC-40 and SAX-3 are not physically associated. UNC-40 associates with SYD-1 and negatively regulates MIG-2, which functions in the SAX-3 pathway. (TIF) [file pgen.1005185.s003.tif]

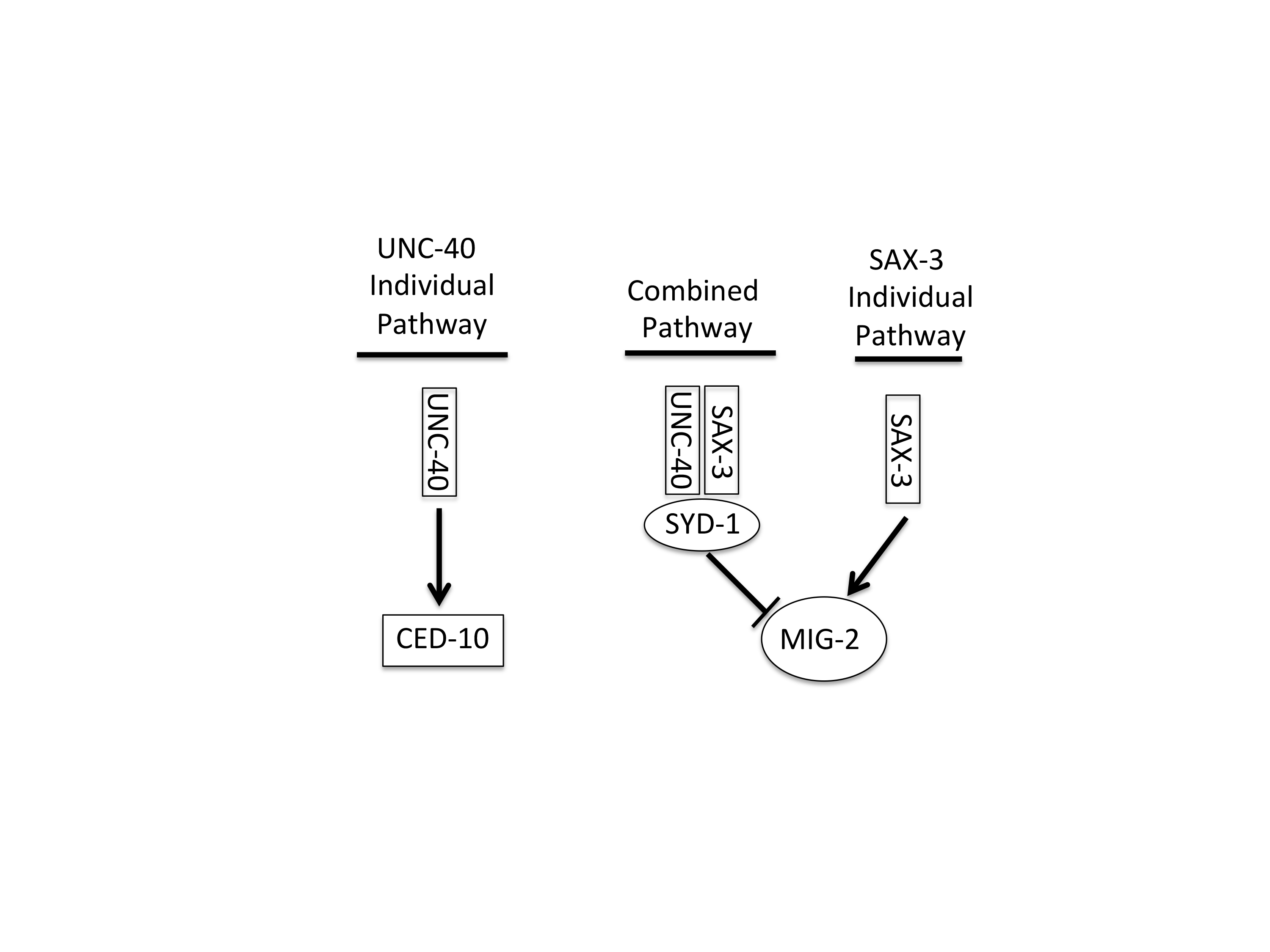

Supplement: S4 Fig — UNC-40 can function individually by activating effectors such as CED-10, MIG-10, UNC-34 and UNC-115. SAX-3 can function individually by activating MIG-2. UNC-40 and SAX-3 can also function in a combined pathway, where they collaborate to regulate MIG-2. SYD-1 is specific to the combined pathway. This example is depicted with the heterodimer model (see S3 Fig), however the same idea could also apply to the cross talk model. (TIF) [file pgen.1005185.s004.tif]
